# The frequency of brain lesion on CT scan in traumatic pediatric that referred to Ayatollah Taleghani Hospital of Kermanshah 2011

**Published:** 2012-11

**Authors:** Saleh Salehi Zahabi, Mahmmod Mehrbakhsh, Kharaman Salehi Zahabi, Shahriar Asgari, Shahnaz Darabi, Karam Ahmadi

**Affiliations:** ^*a*^Kermanshah University of Medical Sciences, Kermanshah, Iran.; ^*b*^Medical University, Kermanshah University of Medical Sciences, Kermanshah, Iran.; ^*c*^Taleghani Hospital , Kermanshah University of Medical Sciences, Kermanshah, Iran.; ^*d*^Baghiatolah University of Medical Sciences, Tehran, Iran.

## Abstract

**Background:**

Brain trauma (BT) is the most common cause of death among children worldwide. In traumatic patient, the skull is the most common involved part. The importance of computed tomography (CT) scan in diagnosis of BT is well established. CT scan is actually a common option for evaluation of patients with cranial trauma. Considering the importance of CT scan in the diagnosis of brain lesions, the present study was aimed to survey the results of brain CT scan in traumatic patient attending the Ayatollah Taleghani Hospital (Kermanshah, Iran) during 2011.

**Methods:**

In this descriptive and cross-sectional study brain CT scan results of 365 patients with head trauma attending the CT scan department of Taleghani Hospital (Kermanshah) during 2011 was evaluated. For data collection, pre-prepared tables containing demographic data, cerebral results, lesion and location of fractures were used. Finally the data were analyzed using descriptive statistics.

**Results:**

The results show that of 907 patients, 557 were male and 350 female. In this study we performed CT scans on 365 traumatic patients, 326(89.31%) cases of 365 patients had normal brain CT scan and 39(10.69%) cases had positive finding including:

3 cases (7.69%) of brain contusion, 5 cases (12.82%) of epidural hematoma, 4 cases (10.26%) of subdural hematoma, 3 cases(7.69%) of subarachnoid hemorrhage, 2 cases (5.13%) of intracranial hemorrhage, 2 cases (5.13%) of intraventricel hemorrhage, and finally 20 cases (51.28%) of skull fracture.

**Conclusions:**

Because of the high percentages of normal CT scan with no clinical indication in this study, it is necessary for clinical physicians to pay enough attention and rethink about performing a brain CT scan in a patient, particularly when they are not sure about the necessity of a performing CT scan. Furthermore, there is a need for further studies managing the current trend among physicians in which brain CT scan is going to be a routine application.

**Keywords:**

Brain CT scan, Pediatric, Trauma, Computed tomography


					Volume 4, Suppl. 1 Nov 2012
Publisher: Kermanshah University of Medical Sciences
URL: http://www.jivresearch.org
Copyright:
Congress of Iranian Neurosurgeons, 14-16 November 2012, Kermanshah, Iran
Paper No. 50
The frequency of brain lesion on CT scan in traumatic
pediatric that referred to Ayatollah Taleghani Hospital of
Kermanshah 2011
Saleh Salehi Zahabi a ,*
, Mahmmod Mehrbakhsh a
, Kharaman Salehi Zahabi b
, Shahriar Asgari c
,
Shahnaz Darabi b
, Karam Ahmadi d
a
Kermanshah University of Medical Sciences, Kermanshah, Iran.
b
Medical University, Kermanshah University of Medical Sciences, Kermanshah, Iran.
c
Taleghani Hospital , Kermanshah University of Medical Sciences, Kermanshah, Iran.
d
Baghiatolah University of Medical Sciences, Tehran, Iran.
Abstract:
Background: Brain trauma (BT) is the most common cause of death among children worldwide. In traumatic patient,
the skull is the most common involved part. The importance of computed tomography (CT) scan in diagnosis of BT is
well established. CT scan is actually a common option for evaluation of patients with cranial trauma. Considering the
importance of CT scan in the diagnosis of brain lesions, the present study was aimed to survey the results of brain CT
scan in traumatic patient attending the Ayatollah Taleghani Hospital (Kermanshah, Iran) during 2011.
Methods: In this descriptive and cross-sectional study brain CT scan results of 365 patients with head trauma
attending the CT scan department of Taleghani Hospital (Kermanshah) during 2011 was evaluated. For data
collection, pre-prepared tables containing demographic data, cerebral results, lesion and location of fractures were
used. Finally the data were analyzed using descriptive statistics.
Results: The results show that of 907 patients, 557 were male and 350 female. In this study we performed CT scans
on 365 traumatic patients, 326(89.31%) cases of 365 patients had normal brain CT scan and 39(10.69%) cases
had positive finding including:
3 cases (7.69%) of brain contusion, 5 cases (12.82%) of epidural hematoma, 4 cases (10.26%) of subdural
hematoma, 3 cases(7.69%) of subarachnoid hemorrhage, 2 cases (5.13%) of intracranial hemorrhage, 2 cases
(5.13%) of intraventricel hemorrhage, and finally 20 cases (51.28%) of skull fracture.
Conclusions: Because of the high percentages of normal CT scan with no clinical indication in this study, it is
necessary for clinical physicians to pay enough attention and rethink about performing a brain CT scan in a patient,
particularly when they are not sure about the necessity of a performing CT scan. Furthermore, there is a need for
further studies managing the current trend among physicians in which brain CT scan is going to be a routine
application.
Keywords:
Brain CT scan, Pediatric, Trauma, Computed tomography
* Corresponding Author at:
Saleh Salehi Zahabi : M.Sc Student in Medical Physics, Faculty of Medicine. Tarbiat Modares University, Tehran, Iran, Tel: 08314263071,
Email: sszahabi@gmail.com, (Salehi Zahabi S.).

				

